# Go Big or go home: a new gene ontology subset that improves plant gene function prediction

**DOI:** 10.1186/s13007-026-01523-8

**Published:** 2026-03-29

**Authors:** Leila Fattel, Carolyn J. Lawrence-Dill

**Affiliations:** 1 Interdepartmental Genetics and Genomics, Iowa State University, Ames, IA 50011 USA; 2 Department of Agronomy, Iowa State University, Ames, IA 50011 USA; 3 Department of Soil and Crop Sciences, Colorado State University, Fort Collins, CO 80523 USA

**Keywords:** Gene ontology, Plants, Gene function, Annotation

## Abstract

**Background:**

The availability of gene function prediction datasets helps researchers to consider possible functions for uncharacterized genes for hypothesis generation, candidate gene prioritization, and many other applications. Many such datasets are based on the Gene Ontology (GO) function graph. For plants this can be problematic because the most specific GO terms available are often derived from the biology of non-plant taxa (e.g., functions specific to nerve function would not seem likely to map to plant biological processes given that plants lack nerves). To balance the need for functional specificity while limiting to functions relevant to plant biology, researchers often limit to the GO Slim plant subset, but, by design, that subset consists of very general terms and limits real utility for, e.g., specific hypothesis generation. Worse yet, sometimes researchers choose to simply throw out terms if they are not relevant to plant biology (rather than traversing the GO graph to select the most specific term in that hierarchy that is compatible with plant biology).

**Results:**

We created GO Big, a Gene Ontology subset type, to improve the biological relevance of gene function predictions for taxon-specific biology applications. GO Big plant subsets retain maximal functional specificity for hypothesis generation while limiting to terms applicable to the biology of plants. In brief, we used a curatorial approach to generate two GO Big subsets, a general subset derived from terms with experimentally validated functions across Viridiplantae species, and a species-specific subset for maize (*Zea mays* ssp. *mays*).

**Conclusion:**

Annotating genes with assignments that better reflect the biology of a taxon can pave the way for more biologically accurate and testable hypotheses for genes of interest. The subsets produced here can help plant biologists limit genome-wide gene function prediction sets to functions possible for plant genes, and the process to generate GO Big subsets is described in detail to enable others to create GO Big subsets for additional taxon sets, including ones for protists, fungi, and other phylogenetic categories.

**Supplementary Information:**

The online version contains supplementary material available at 10.1186/s13007-026-01523-8.

## Introduction

The development of the bio-ontologies has been integral for the structured representation of biological knowledge to enable computational approaches [1]. Of note is the regularly updated Gene Ontology (GO) created and maintained by the Gene Ontology Consortium [2, 3]. The GO is a directed acyclic graph (DAG) of standardized vocabulary terms that represents the functional characteristics of genes and gene products [4]. GO has been used by researchers to predict the function of a gene of interest, to find common functions between genes located in one or more organisms, to evaluate the abundant functions in gene expression studies, and to hypothesize possible protein-protein interactions, to name a few [5]. There are three categories of GO terms: Biological Process (BP), Molecular Function (MF), and Cellular Component (CC) [2]. The terms (nodes) in the GO knowledgebase are connected through relations (edges), such as “is a”, “part of”, “regulates”, “positively regulates”, “negatively regulates”, and more [3]. In addition, GO annotation assignments are supported by evidence codes so that the logic for how a term was assigned is explained. These evidence codes are classified under six general categories: experimental, phylogenetic, computational, author statements, curatorial statements, and automatically generated annotations [3]. Initially, GO terms were derived from three model organism databases [2]: Saccharomyces Genome Database (SGD) [6], FlyBase [7], and Mouse Genome Informatics (MGI) [8]. Since then, several collaborators from various model organism and protein databases have joined in the efforts of the GO consortium to develop and expand the framework to include several animal, plant, fungal, bacterial, and viral gene function annotations [3, 9]. As of April 2024, there were 42,255 GO terms in use [10].

Gene function prediction tools provide researchers with gene function assignments that can help them to identify genes responsible for phenotypes of interest, to discover potential drug targets, and to find underlying causes of diseases [11]. An example of a plant-specific gene function prediction pipeline is the Gene Ontology Meta Annotator for Plants (GOMAP), which produces high-coverage and reproducible GO-based functional annotation datasets [12]. However, a problem for gene function prediction in plants is the lack of some specific GO terms related to plant function, coupled with the presence of some very detailed gene function term assignments derived from animal, fungi, and microorganisms. This results in less accurate annotation assignments for plants [13]. For example, a study we conducted discussed that some plant genes were assigned with GO terms like “animal organ development” (GO:0048513), while also being annotated with plant-specific functions [14].

GO slims are reduced and manageable subsets of the GO that contain terms describing gene functions in a broad manner [9, 15]. Several GO slims currently exist and can be found in the GO database (https://geneontology.org/docs/download-ontology/#subsets) [16]. Subsets can be species-specific, such as that for the fungus *Candida albicans* (GO Slim candida) or even kingdom-specific, such as that for plants (GO Slim plant) [5]. These high-level GO terms are tagged within the GO DAG to allow the display of (and functional limitation to) each subset to which the term belongs [3]. The main purpose of a GO slim is to represent and map gene function annotations to a summary of general biological functions [17]. The small number of high-level GO terms that are members of each GO Slim subset were selected as a set of terms that would be most effective at capturing all categories of functions derived from all annotated genes of the taxa. GO slims are often used in gene expression studies to highlight over- and under-represented functions shared by a group of genes in one or more organism [18].

When it comes to gene function prediction, annotating with a GO term that is as specific as possible, while allowing for incorrect assignments, can be beneficial as these annotations can become starting points for generating and testing hypotheses for genes of unknown functions. However, some plant biology researchers are only interested in plant-possible functions. So far, there had been two GO-based approaches to help with that: (1) mapping against the plant GO slim subset to get higher-level, biologically relevant functions, and (2) relying on the taxon constraints set by the GO Consortium through “only in taxon" and “never in taxon" relationships allocated to some GO terms, a quality control method used by curators to detect incorrect gene function annotations [19]. Here, we discuss a third approach that is evidence-based and dependent on the taxa of interest. The GO Big subsets maximize taxonomic unit specificity while limiting to terms relevant to the biology of the included taxa to assist with automated gene function prediction analyses. We also describe the methods used to create a species-specific subset (e.g., GO Big maize) and a more general green plants subset (GO Big plant).

## Methods

Creating GO subset files can be time- and labor-intensive. In this section, we demonstrate creating the GO Big subsets, “GO Big maize" and “GO Big plant", using two methods. They both consist of the following steps: accumulating GO terms assigned with experimental evidence codes, collecting the ancestors of these GO terms, tagging the GO terms of interest as members of the GO Big subset, and creating the GO Big subset file.

### The GO Big maize subset (as a species-specific example)

GO terms assigned through manual curation from experimental literature of the *Zea mays* ssp. *mays* genome were gathered using the Gene Ontology Annotation (GOA) database [20]. The filters were chosen as follows:

- Taxon: *Zea mays* (taxon ID: 4577) and exact match.
- GO terms: For each GO category at a time, BP, MF, and CC (selected under the “Aspect” filter), their respective GO terms belonging to the GO Slim plant subset were added as identifiers (BP: 45; MF: 25; CC: 24), and the option “use these terms as a GO slim” was selected to find GO terms that map back to the plant subset. Alternatively, one can select all GO categories under the “Aspect” filter at the same time and take advantage of the “select terms from GO slim” option under “GO terms” by choosing “goslim_plant". However, this was not done here because the output files can be too large and we settled on splitting them by GO aspect instead.
- Evidence: To avoid collecting plant genes with errant function assignments, only experimental evidence codes were selected. Experimental evidence codes indicate that there is direct evidence within the literature that supports the annotation of a gene. The experimental evidence codes - along with their child terms - chosen were ECO:0000352 (evidence used in manual assertion), ECO:0000269 (experimental evidence used in manual assertion), ECO:0000314 (direct assay evidence used in manual assertion), ECO:0000315 (mutant phenotype evidence used in manual assertion), ECO:0000316 (genetic interaction evidence used in manual assertion), ECO:0000353 (physical interaction evidence used in manual assertion), ECO:0000270 (expression pattern evidence used in manual assertion), ECO:0006056 (high throughput evidence used in manual assertion), ECO:0007005 (high throughput direct assay evidence used in manual assertion), ECO:0007001 (high throughput mutant phenotypic evidence used in manual assertion), ECO:0007003 (high throughput genetic interaction phenotypic evidence used in manual assertion), and ECO:0007007 (high throughout expression pattern evidence used in manual assertion).

Once the filters were applied, the files were downloaded and the annotations under the “GO term” column were looked up using QuickGO [21] for manual collection of these terms and their ancestors all the way up to the root term. While collecting all these terms, their descriptions were inspected to make sure they had functions applicable in the context of plants. After GO term collection, Protégé [22] was used to tag these terms as members of the GO Big maize subset using the go.owl file release “2023-10-09”. Robot [23] was then used to create the gobig_maize.owl file (Web Ontology Language—a file format for knowledge representations like ontologies) by selecting and filtering for the terms tagged as part of the subset. Subsequently, the gobig_maize.obo file (Open Biomedical Ontologies—a file format for representing ontologies and controlled vocabularies) was also created.

### The GO Big plant subset

To create the GO Big subset for green plants (Viridiplantae), we included more species to capture diverse plant functions. All GO annotations with experimental evidence codes assigned to plant species that are descendants of Viridiplantae (taxon ID: 33090) were collected from the GOA database. Unlike the GO Big maize subset, we did not filter the GO terms using the GO Slim plant subset as identifiers so that all GO annotations with manually assigned evidence codes would be captured.

**Fig. 1:**
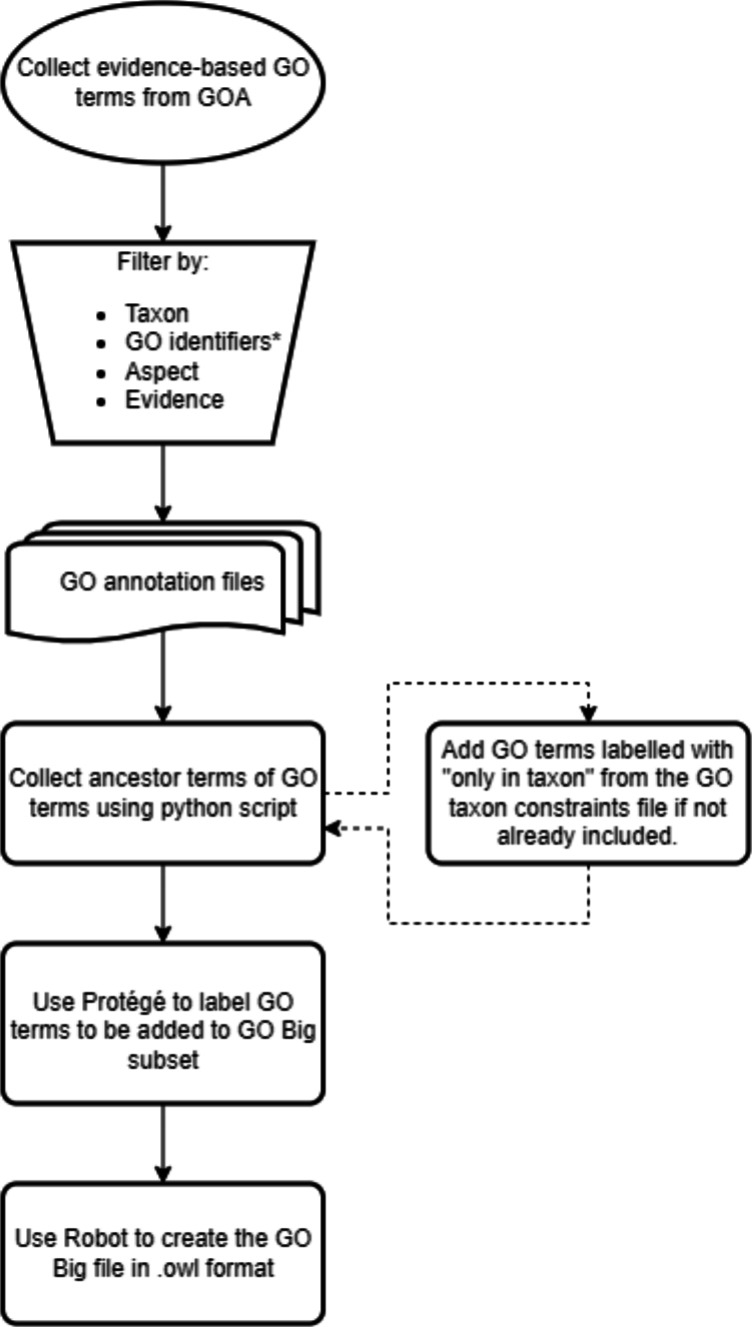
Workflow for creating a GO Big subset. *Oval* represents start, inverted trapezoid represents a manual process, *curved rectangle* represents multiple documents, and *rectangles* represent tasks with outputs. * symbolizes only in GO Big maize. *Dashed arrows* represent the additional process included for GO Big plants

The generation of the GO Big plant subset (gobig_plant.owl and gobig_plant.obo files) was similar to the method described for GO Big maize. To automate the collection of the GO ancestors, we created python scripts using the GOATOOLS package [24]. Here, the go.owl file release “2024-04-24” was used to manually tag the terms belonging to the GO Big plant subset in Protégé. Additionally, using the go_taxon_constraints.owl file provided by the GO Consortium [25], any GO term labelled with “only in taxon" for the class Viridiplantae that was not experimentally assigned to a plant gene was added to the subset (as well as its ancestor terms) as a way to further capture more plant functions in this subset. The method is summarized in Fig. 1. All the files and scripts described here can be found in our GitHub repository [26].

### GO overrepresentation assessment

The Biological Network Gene Ontology tool (BiNGO) [27] in Cytoscape [28] was used to perform GO overrepresentation assessment using the GO Slim plant subset, the GO Big maize subset, the GO Big plant subset, and the full GO DAG. This tool was selected because it allows a user to upload a custom GO subset. For the gene subset used as an input, 27 SET domain-containing gene models in the *Z. mays* B73v5 genome were selected from MaizeGDB [29] to perform GO overrepresentation analysis on. The list of gene names (and gene IDs) are as follows: *sdg122* (Zm00001eb005310), *sdg103* (Zm00001eb042640), *sdg123* (Zm00001eb048280), *sdg140* (Zm00001eb069580), *sdg104* (Zm00001eb075120), *sdg107* (Zm00001eb076530), *sdg102* (Zm00001eb082920), *sdg137* (Zm00001eb096860), *sdg110b* (Zm00001eb097830), *sdg119* (Zm00001eb098590), *sdg106* (Zm00001eb104550 and Zm00001eb104560), *sdg116* (Zm00001eb104980), *sdg113* (Zm00001eb150160), *sdg115* (Zm00001eb158060), *sdg108* (Zm00001eb171790), *sdg117* (Zm00001eb188560), *sdg130* (Zm00001eb216690), *sdg129* (Zm00001eb230520), *sdg111* (Zm00001eb291310), *sdg101* (Zm00001eb303540), *sdg128* (Zm00001eb308480), *sdg110a* (Zm00001eb309540), *sdg40* (Zm00001eb320090), *sdg105* (Zm00001eb352310), *sdg118* (Zm00001eb363830), and *sdg127* (Zm00001eb406010). The parameters in BiNGO were set to as follows: Assess - *Overrepresentation*, Multiple testing correction - *Benjamini & Hochberg False Discovery Rate (FDR) correction*, Significance level - *0.05*, Categories to be visualized - *Overrepresented categories after correction*, Reference set - *Use whole annotation as reference set*, Ontology file - *GOSlim_Plants*, *Custom/gobig_maize.obo*, *Custom/gobig_plant.obo* or *GO_Full*, and Organism/Annotation - *maize B73v5 annotations file generated using GOMAP* [30]. Figures showing the statistically overrepresented GO terms network based on the four ontology files were generated.

## Results

### The GO Big subsets

Because the GO Big plant subset includes GO terms derived from more than one plant, it is (approximately 11X) larger than the GO Big maize subset, as seen in Table 1. The GO Big plant subset has a total of 10,611 GO terms that includes 6,236 BP, 1087 CC, and 3,288 MF terms. Meanwhile, the GO Big maize subset has a total of 1,003 GO terms that contain 599 BP, 88 CC, and 316 MF terms.

### General comparison of the GO Big subsets with the full GO DAG and GO Slim plant subset

Both the GO Big subsets contain more terms than the GO Slim plant subset. Although the GO Big maize subset (1003 GO terms) includes terms derived from experiment-based annotations from only one plant, it is still approximately 10X larger than the GO Slim plant subset (97 GO terms). At the same time, the GO Big plant subset (10,611) contains around 109X the GO terms included in GO Slim plant. On the other hand, GO Big maize and GO Big plant only cover $\sim {2}$% and $\sim {25}$% of the entire GO corpus, respectively. A full comparison of the number of GO terms by aspect is shown in Table 1. To demonstrate the impact of interpreting gene function based on GO Big on functional annotation, we provide an example in the next section.

**Table 1:** Number of GO terms by GO aspect in each GO set

GO set	GO aspect [count (*)]			
	Biological process	Cellular component	Molecular function	All
GO Big plant	6236 (58.77%)	1087 (10.24%)	3288 (30.99%)	10,611
GO Big maize	599 (59.72%)	88 (8.77%)	316 (31.51%)	1003
GO Slim plant	46 (47.42%)	25 (25.78%)	26 (26.80%)	97
GO release "2023-10"	27,538 (64.28%)	4060 (9.48%)	11,239 (26.24%)	42,837
GO release "2024-04"	27,047 (64.01%)	4057 (9.60%)	11,151 (26.39%)	42,255

*Parenthetical numbers reflect percentage of terms by aspect out of all terms available in the respective set

### Demonstration: non-plant annotations for a plant gene

The SET (Su(var)3-9, Enhancer-of-zeste and Trithorax) domain is often found in various histone methyltransferases that play a role in epigenetic modifications [31]. In plants, histone methyltransferases play a role in several biological processes, including morphogenesis, hormone regulation, flowering time, shoot and root branching, seed development, circadian cycle, and some stress responses [31, 32]. A recent study reported that the SET domain gene 130 (*sdg130*) is a candidate gene involved in the regulation of vegetative phase change in maize upon jasmonic acid treatment [33]. The GO annotations for *sdg130* (gene ID Zm00001eb216690) in the maize B73v5 genome [30] using the Gene Ontology Meta Annotator for Plants (GOMAP) [12] are shown in Table 2. The annotations mainly describe functions relating to histone methylation and transcription processes. However, of the 28 GO terms, four describe non-plant functions: “skeletal muscle myosin thick filament assembly” (GO:0030241), “sarcomerogenesis” (GO:0048769), “cardiac myofibril assembly” (GO:0055003), and “skeletal muscle organ development” (GO:0060538). A common approach to deal with terms describing non-plant functions is to ignore them. However, these errant GO annotations may be useful.

**Table 2:** GO term annotations for the maize B73v5 *sdg130* gene assigned by GOMAP

GO term	GO aspect*	GO description
GO:0000122	BP	Negative regulation of transcription by RNA polymerase II
GO:0006325	BP	Chromatin organization
GO:0006351	BP	Transcription, DNA-templated
GO:0008285	BP	Negative regulation of cell population proliferation
GO:0010452	BP	Histone H3-K36 methylation
GO:0010629	BP	Negative regulation of gene expression
GO:0018026	BP	Peptidyl-lysine monomethylation
GO:0018027	BP	Peptidyl-lysine dimethylation
GO:0030241	BP	Skeletal muscle myosin thick filament assembly†
GO:0034645	BP	Cellular macromolecule biosynthetic process
GO:0043516	BP	Regulation of DNA damage response, signal transduction by p53 class mediator
GO:0048769	BP	Sarcomerogenesis†
GO:0051276	BP	Chromosome organization
GO:0051568	BP	Histone H3-K4 methylation
GO:0055003	BP	Cardiac myofibril assembly^†^
GO:0060538	BP	Skeletal muscle organ development^†^
GO:2000113	BP	Negative regulation of cellular macromolecule biosynthetic process
GO:0005634	CC	Nucleus
GO:0005694	CC	Chromosome
GO:0005829	CC	Cytosol
GO:0016021	CC	Integral component of membrane
GO:0000993	MF	RNA polymerase II complex binding
GO:0002039	MF	p53 binding
GO:0008270	MF	Zinc ion binding
GO:0017022	MF	Myosin binding
GO:0042800	MF	Histone methyltransferase activity (H3-K4 specific)
GO:0044877	MF	Protein-containing complex binding
GO:0046975	MF	Histone methyltransferase activity (H3-K36 specific)

*BP biological process, *CC* cellular component, *MF* molecular function

^†^GO term descriptions that are not applicable as plant function descriptions

To evaluate if errant GO terms can provide useful insights while staying relevant to plant biology, we mapped the 28 GO annotations to the GO Slim plant, GO Big maize, and GO Big plant subsets using GOTermMapper [9, 34] to identify information loss in each subset (see Additional File 1). Mapping resulted in 21 GO terms (13 BP, 4 MF, and 4 CC) using the GO Slim plant subset, 103 GO terms (75 BP, 16 MF, and 12 CC) using the GO Big maize subset, and 149 GO terms (105 BP, 32 MF, and 12 CC) using the GO Big plant subset. In general, the terms from the GO Slim plant subset describe high-level processes like “protein metabolic process” (GO:0019538) and “multicellular organism development” (GO:0007275), and functions like “protein binding” (GO:0005515) and “transferase activity” (GO:0016740). With the GO Big maize subset, however, the GO term descriptions start becoming more specific, and include processes relating to transcription (“regulation of DNA-templated transcription” (GO:0006355); “chromosome organization” (GO:0051276); “gene expression” (GO:0010467)), and morphogenesis (“anatomical structure morphogenesis” (GO:0009653); “system development” (GO:0048731); “tissue development” (GO:0009888)). Both of which are biological processes that *sdg130* has been reported to be involved in as described previously. In addition, cytoskeleton-related GO terms, including “cytoskeleton organization” (GO:0007010) and “actin cytoskeleton organization” (GO:0030036) were detected, both of which are ancestor terms for “sarcomerogenesis” (GO:0048769), one of the errant GO annotations. As expected, using the GO Big plant subset resulted in even more specific functions, among which that describe histone methylation, such as “histone H3 methyltransferase activity” (GO:0140938) and “protein methylation” (GO:0006479), presenting more biologically meaningful predictions of the various roles of *sdg130* in *Z. mays* in comparison to the GO Slim plant subset, while also making good use of non-plant function descriptions without fully incorporating them.

**Fig. 2:**
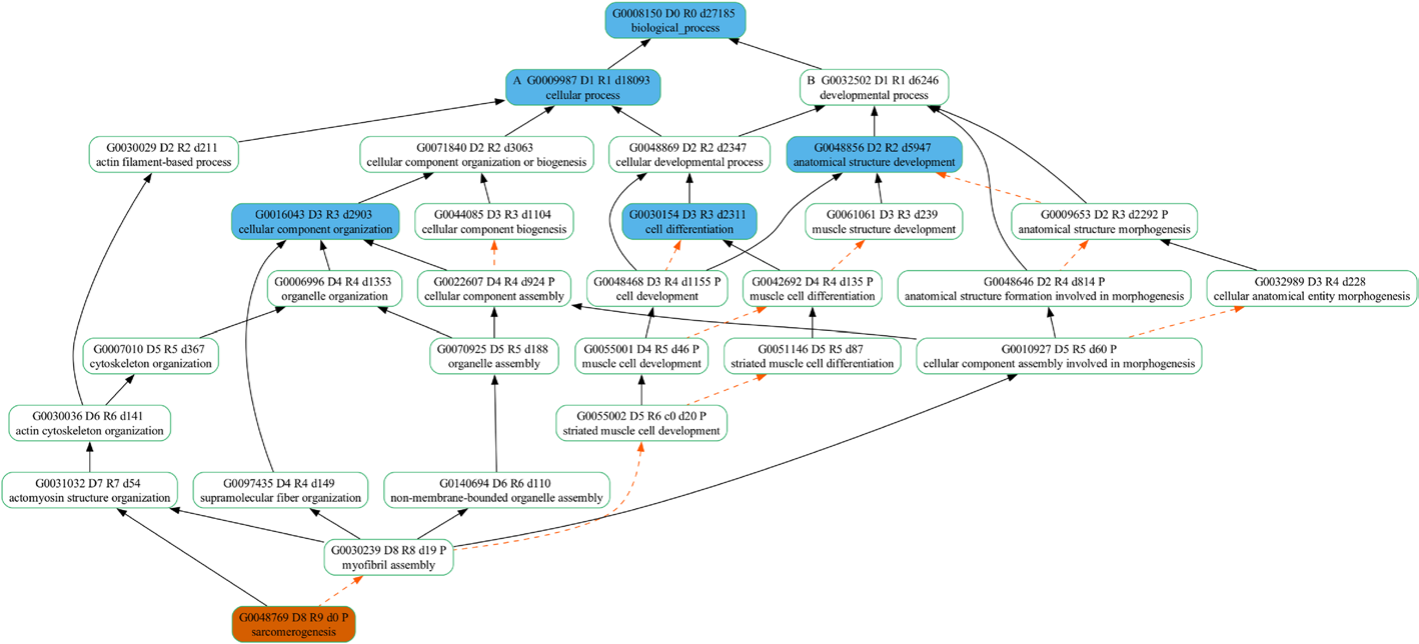
Mapping “sarcomerogenesis" to the GO Slim plant subset. Terms appear in *oval boxes*. *Orange* fill represents the GO term assigned. *Blue* fill represents GO terms included in the GO Slim plant subset. Relationships between terms are shown as *arrows*. *Solid black arrows* denote “is a" relationships, while *dashed arrows* in *orange* denote “part of" relationships. In each *oval box*, the first line is the GO term header. Term identifiers are formatted as G#######. Letters A and B prepending the term identifier demarcate the highest-level ancestors of the term. The letter D prepends a number indicating the maximum distance from the root term (biological process) to the child term using only “is a" relationships; R is the maximum distance from the root term to the child term using both “is a" and “part of" relationships; d is the descendants count

**Fig. 3:**
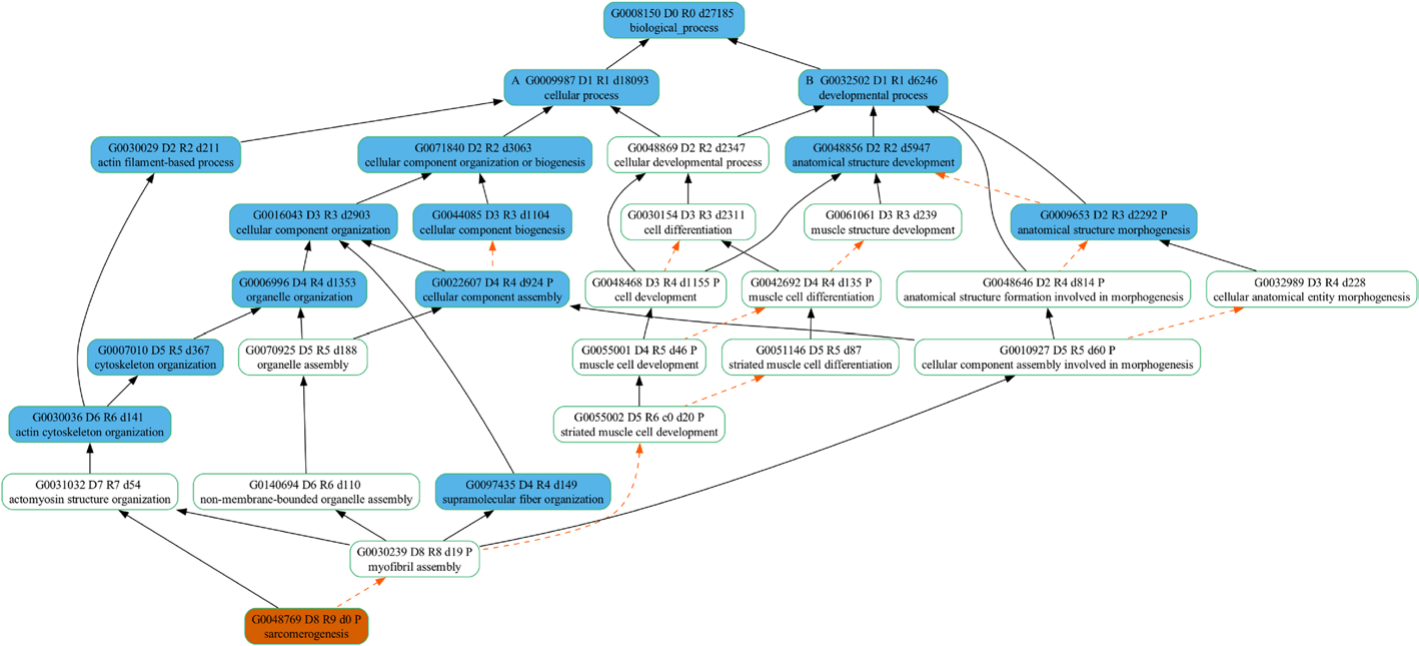
Mapping “sarcomerogenesis" to the GO Big maize subset. Terms appear in *oval boxes*. *Orange* fill represents the GO term assigned. *Blue* fill represents GO terms included in the GO Big maize subset. *Solid black arrows* denote “is a" relationships, while *dashed arrows in orange* denote “part of" relationships. In each *oval box*, the first line is the GO term header. Term identifiers are formatted as G#######. Letters A and B prepending the term identifier demarcate the highest-level ancestors of the term. The letter D prepends a number indicating the maximum distance from the root term (biological process) to the child term using only “is a" relationships; R is the maximum distance from the root term to the child term using both “is a" and “part of" relationships; d is the descendants count

**Fig. 4:**
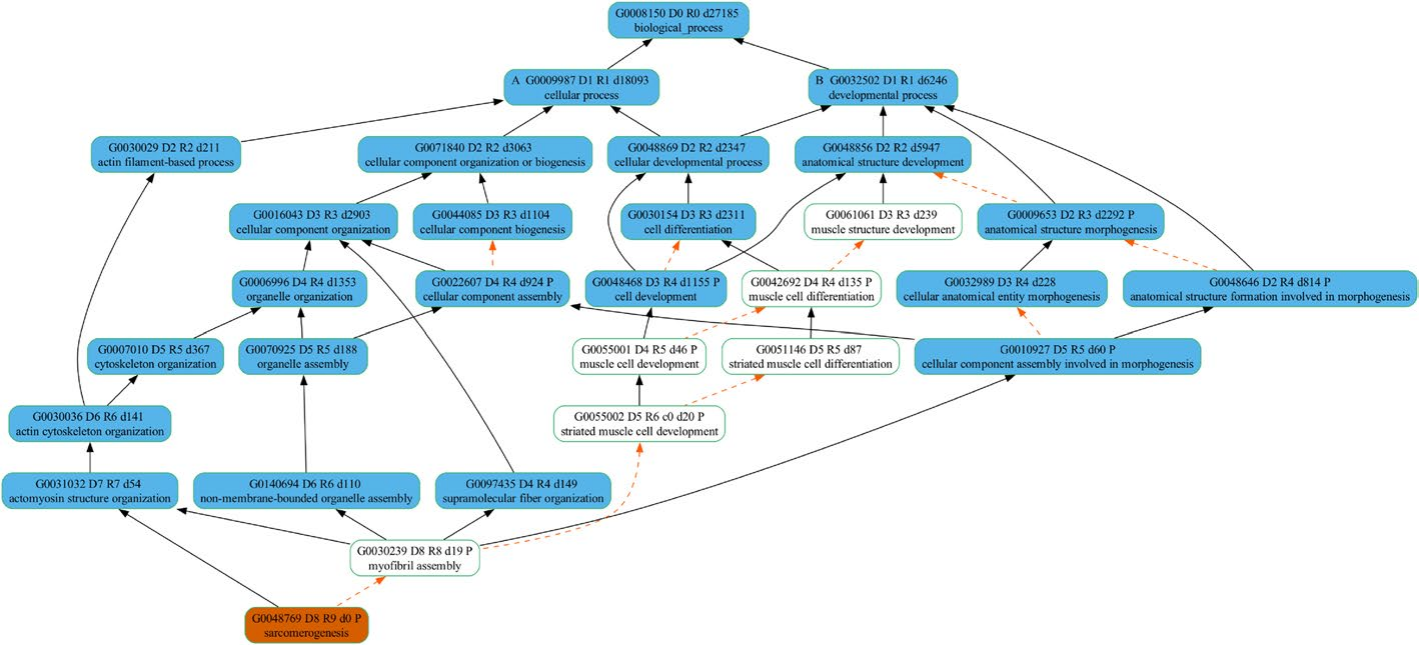
Mapping “sarcomerogenesis" to the GO Big plant subset. Terms appear in *oval boxes*. *Orange* fill represents the GO term assigned. *Blue* fill represents GO terms included in the GO Big plant subset. *Solid black arrows* denote “is a" relationships, while *dashed arrows in orange* denote “part of" relationships. In each *oval box*, the first line is the GO term header. Term identifiers are formatted as G#######. Letters A and B prepending the term identifier demarcate the highest-level ancestors of the term. The letter D prepends a number indicating the maximum distance from the root term (biological process) to the child term using only “is a" relationships; R is the maximum distance from the root term to the child term using both “is a" and “part of" relationships; d is the descendants count

For demonstration purposes, we mapped one of the errant GO terms, “sarcomerogenesis” (GO:0048769), to the GO Slim plant (Fig. 2), GO Big maize (Fig. 3), and GO Big plant (Fig. 4) subsets to show the ancestor terms derived, as well as the depth (the length of the longest path from the top) of the most specific GO term in each case. In Fig. 2, the GO Slim plant resulted in only four high-level GO terms and a maximum depth of three (GO:0016043 and GO:0030154) in the DAG. Although the ancestor terms obtained from the GO Slim plant subset do give a general idea that the possible functions of *sdg130* include structure development and cellular component organization, the GO Big maize subset (Fig. 3) captured 13 ancestor GO terms that cover more processes relating to morphogenesis and, more specifically, the actin filament component of the cytoskeleton with a maximum depth of six (GO:0030036). Finally, the GO Big plant subset (Fig. 4) had the highest coverage of a total of 22 ancestor GO terms, with a maximum depth of seven (GO:0031032). In addition to the observed differences in coverage for the ancestral terms when mapping “sarcomerogenesis” (GO:0048769) to the three GO subsets, the mappings demonstrate the derivation of alternative, more biologically meaningful GO term assignments from each subset in the place of the errant term. The most specific ancestor term along each path of the respective DAG is selected as the replacement GO term from each subset. To illustrate, the GO term annotations replacing the errant term “sarcomerogenesis” (GO:0048769) assigned to *sdg130* are listed in Table 3. Using GO Slim plant resulted in three replacement annotations (GO:0048856, GO:0030154, and GO:0016043), while using GO Big maize resulted in four replacement annotations (GO:0009653, GO:0022607, GO:0097435, and GO:0030036), and using GO Big plant resulted in five replacement annotations (GO:0010927, GO:0048468, GO:0097435, GO:0140694, and GO:0031032). Both GO Big subsets resulted in more specific annotations compared to the GO Slim subset, as well as more relevant and biologically meaningful GO terms.

**Table 3:** GO term annotation assignments replacing “sarcomerogenesis” (GO:0048769) for the maize B73v5 *sdg130* gene from each GO subset

GO slim plant		GO big maize		GO big plant	
GO term	GO description	GO term	GO description	GO term	GO description
GO:0048856	Anatomical structure development	GO:0009653	Anatomical structure morphogenesis	GO:0010927	Cellular component involved in morphogenesis
GO:0030154	Cell differentiation	GO:0022607	Cellular component assembly	GO:0048468	Cell development
GO:0016043	Cellular component organization	GO:0097435	Supramolecular fiber organization	GO:0097435	Supramolecular fiber organization
		GO:0030036	Actin cytoskeleton organization	GO:0140694	Non-membrane-bounded organelle assembly
				GO:0031032	Actomyosin structure organization

Although the GO Big plant subset covers more terms describing plant functions and processes than the GO Big maize subset, some errant terms were still somehow incorporated. Upon further analysis of the GO terms that resulted from mapping the 28 GO term annotations (Additional File 1), two terms from the GO Big plant subset stand out: “animal organ development” (GO:0048513) and “circulatory system development” (GO:0072359). To investigate the reason behind the incorporation of these terms (even when only selecting experiment-based evidence codes), we discuss the two examples below.

#### Example 1: incorporation of GO terms of non-plant functions in a plant subset

To identify the cause of the incorporation of the non-plant function GO term “animal organ development” (GO:0048513), which indeed has not been experimentally annotated to any Viridiplantae gene in the GOA database, we inspected the presence of any of its child terms in the GO Big plant subset. Since the subset was built in a manner that preserves the structure of the GO DAG, any GO term added to the subset will have its ancestor terms included there. Indeed, the term “mesenchyme migration” (GO:0090131) has led to the integration of “animal organ development” (GO:0048513) in the GO Big plant subset as an ancestor term. The child term itself appears problematic in this context as the mesenchyme refers to a population of multipotent cells found in animal models [35]. Interestingly, this term has been manually assigned to the *Z. mays* gene *B4G1E1* (UniProtKB:B4G1E1). Further investigation in the literature has shown that there are studies that report plant products and bioactive compounds playing a role in mesenchymal stem cell proliferation and differentiation [36, 37]. Although the GO term itself is not representative of a function that is present in plant physiology, it seems to reflect a function that represents regulatory interactions between plants and other species. This highlights the importance of inspecting seemingly errant GO terms assigned to plant gene products from a perspective that considers plant-non-plant interactions.

Another child term for the GO term “animal organ development” (GO:0048513) found in our GO Big plant subset is “interkinetic nuclear migration” (GO:0022027). The term “interkinetic nuclear migration” itself has been first used to describe nuclear movements observed during neural proliferation in vertebrates [38]. Although the term does not seem applicable in the context of plants and has been labelled with a taxon constraint of only being in the phylum Chordata, nuclear migration events have been observed in plants in processes like fertilization, shaping of elongated nuclei in root hairs, and plant-microbe interactions [39–41]. Indeed, the term “nuclear migration” (GO:0007097) has been assigned to genes in *Zea mays*, *Arabidopsis thaliana*, *Manihot esculenta*, and *Oryza sativa* in the GOA database. Upon further inspection, the GO term “interkinetic nuclear migration” (GO:0022027) is shown to be manually assigned only to a *Klebsormidium nitens* gene product (UniProtKB:A0A1Y1ICV9) in Viridiplantae in the GOA database. However, on the UniProt database [42], the annotation status of this protein seems to be unreviewed, and computer predicted. Since the GO terms in the GO Big plant subset were collected based on experimental and manual curation, this suggests that some gene annotations may need to be reviewed in terms of the type of evidence code assigned.

#### Example 2: incorporation of GO terms of plant functions with non-inclusive ancestors

To investigate the reason behind the presence of the GO term “circulatory system development” (GO:0072359) in the GO Big plant subset, we also looked at the existence of any of its child terms in our subset. In fact, we were able to identify the GO term “regulation of vasculature development” (GO:1901342) as its most specific child term along the path of the DAG in the subset. In the GOA database, this GO term has been assigned to the *ABERRANT PANICLE ORGANIZATION* (*APO1*) gene in *Oryza sativa* ssp. *indica* and *japonica* (UniProtKB:B8B183 and UniProtKB:Q655Y0). It has been reported that this gene plays a role in vascular bundle systems development [43]. Inspecting the parent terms of “regulation of vasculature development” (GO:1901342) shows that it has a “regulates” relationship with “vasculature development” (GO:0001944). Interestingly, although “vasculature development” is a biological process that does realistically exist in plants, it has been labelled with a taxon constraint of only being in the subphylum Vertebrata. In turn, “vasculature development” (GO:0001944) is labelled as “part of” the ancestor GO term “circulatory system development” (GO:0072359). Biologically speaking, the term “vasculature development” indicates a more species-inclusive process than the term “circulatory system development”. This indicates the presence of GO terms with ancestors representing less species-inclusive functions, making the higher-level terms less broad. This suggests that some high-level GO terms need to be reviewed either to become more taxon-neutral than their child terms or an alternate path in the DAG needs to be established with non-animal equivalent functions.

### Demonstration: GO overrepresentation assessment using the GO Big subsets

In the previous section, we illustrated the advantages of the GO Big subsets in the context of gene function assignment. In addition, GO terms are often employed in gene enrichment studies. To demonstrate the suitability of GO Big subsets in that regard, we expanded the SET domain-containing gene example used in the previous section into a list of 27 *sdg* gene models from the maize B73v5 genome and performed a GO statistical overrepresentation analysis in BiNGO using the GO Big subsets. For comparison, we ran the same analysis using the GO Slim plant subset and the full GO DAG as standards. The resulting GO networks that contain the statistically overrepresented GO terms and their ancestors based on each ontology are as follows: 22 GO terms for the GO Slim plant subset, 71 GO terms for the GO Big maize subset, 236 GO terms for the GO Big plant subset, and 303 GO terms for the full GO DAG (see Additional File 2). For the GO Slim plant subset, 14 GO terms were statistically significant (*p* < 0.05) and included high-level terms like “transferase activity" (GO:0016740), “protein modification process" (GO:0036211), “nucleus" (GO:0005634), and “epigenetic regulation of gene expression" (GO:0040029), which are consistent with the functions of histone methyltransferases (see Additional File 3). Interestingly, the GO Big maize subset resulted in 64 statistically significant GO terms with more specific descriptions in all three GO categories for the 27 *sdg* gene models, including “chromosome organization" (GO:0051276), “gene expression" (GO:0010467), “chromosome, centromeric region" (GO:0000775), and “zinc ion binding" (GO:0008270) (see Additional File 4). Furthermore, of the 236 GO terms, 155 were identified as overrepresented when using the GO Big plant subset. The terms contained even more specific descriptions for histone methyltransferases like “protein-lysine N-methyltransferase activity" (GO:0016279), “methyl-CpG binding" (GO:0008327), “histone binding" (GO:0042393), and “chromatin remodeling" (GO:0006338) (see Additional File 5). Although the full GO DAG resulted in 211 statistically overrepresented GO terms, the most among the four ontologies, the results included GO terms that described animal functions like “skeletal muscle organ development" (GO:0060538) and “cardiac myofibril assembly" (GO:0055003) (see Additional File 6). In general, using the GO Big subsets for GO overrepresentation assessment provided more information about the enriched functions among the list of genes than the GO Slim plant subset, and reduced the appearance of errant terms compared to using the full GO DAG.

## Discussion

### The new GO Big DAG

We created a new type of GO subset, the GO Big subset, to improve the specificity of gene function prediction in plants. The main purpose of this type of subset is to contain biologically meaningful GO terms for one or more species of interest for accurate and precise prediction tools. Although slim GO subsets have been created and are often used, the purpose of GO Slim subsets is to give a generalized view of the functions represented in a group of genes for applications like high-throughput expression studies or annotation coverage evaluation [17, 44, 45]. The purpose of GO Big subsets – to get the most specific GO term possible without compromising its biological validity within a taxon - requires that the maximum possible specificity for the taxon be annotated. The biggest inspiration for the need of these GO Big subsets was the plant community. A common struggle in plant genomics is the annotation of some of the genes with errant functions. This problem is mainly due to the automatic propagation of these GO terms among genomes by computational pipelines [46].

As seen in the first example detailed in our results, not only did both GO Big subsets produce more detailed GO descriptions for the maize gene *sdg130* as a histone methyltransferase compared to the GO Slim plant subset, but the originally assigned errant annotations were mostly substituted with more relevant and biologically valid functions within the maize species. However, although the GO Big subsets would present more specific plant function annotations than the GO Slim plant subset, there are some limitations that must be considered, as shown in the examples discussed in the previous section:

1. The GO Big subsets discussed in this study are an initial attempt to create a new type of species-specific and/or clade-specific subset. Although our subsets include taxon-neutral and plant-specific GO terms, creating a species-specific subset, like GO Big maize, can still lack common plant-specific functions and require expansion by including GO annotations from a broader clade to encapsulate diverse functions. The more species included, the better the coverage of plant functions, as seen between the GO Big maize and GO Big plant subsets.
2. The methods used to create these subsets are simple and user-friendly so that any researcher would be able to create one for their species of interest. However, this method needs to be coupled with manual expert and curatorial knowledge. For example, in the case of some plants, compared to their large number of genes, experiment-based annotations are scarce or nonexistant [47]. This phenomenon was observed for the species *Pinus lambertiana*, *Solanum pennellii*, *Vaccinium corymbosum L.*, and *Coffea canephora*. Regions in the DAG consisting of GO terms describing uniquely plant processes, functions and components could be included in the GO Big plant subset, regardless of evidence codes. For example, the terms “extrinsic component of lumenal side of plastid thylakoid membrane" (GO:0035450) and “extrinsic component of stromal side of plastid inner membrane" (GO:0035454) clearly represent cellular components found in plants. Those terms were not annotated to any Viridiplantae gene - not even with an IEA evidence code - and were therefore not part of our subset. Additionally, the term “halotropism" (GO:0170002), a process that describes the tropic movement of roots in response to sodium [48], is also missing from our GO Big plant subset due to the lack of its assignment to any plant genes. Other examples of processes found in plants include “seed dehydration" (GO:1990068), “plant gross anatomical part developmental process" (GO:0160109), and “detection of parasitic plant" (GO:0002243). Perhaps, in this case, the availability of both an experimentally derived GO Big plant subset and another separate expanded GO Big plant subset that includes plant and taxon-neutral functions regardless of the evidence type may allow for uniquely useful applications in plant biology research.
3. There is an issue of research imbalance when it comes to genes of interest in plant and animal species [11]. Many researchers focus on studying genes of known functions when there still is a significant percentage of genes of unknown function in eukaryotic genomes [49]. Overcoming this bias may lead to the discovery of unique functions and the creation of new GO term annotations better suited to describe these novel processes, which could be electronically propagated to genes in other genomes, allowing the generation of new hypotheses to test, and ultimately leading to more experimentally verified annotations.
4. Another consideration is that the GO DAG itself (and therefore the GO Big plant subset) lacks good portrayal of diverse plant functions and may need to be expanded in some areas to include those unique plant biology aspects [13]. Most plant functions in the GO DAG were likely created for the model organism *Arabidopsis thaliana*. This single source for plant functions could lead to occurrences where the assignment of unusual functions is due to the absence of related plant functions in the GO graph. On the other hand, it is also worth noting that an unconventional function assigned to a plant gene may not be a false prediction, but simply one that has not yet been observed in plants or discovered experimentally.
5. As seen in this study, a few GO terms that appear errant were actually assigned to some plant genes manually, which resulted in their incorporation in our GO Big plant subset. Upon further inspection, these GO terms were mostly reflecting relationships, mainly regulatory interactions, between plants and other organisms. This underscores the importance of considering the context of the errant assigned annotation before excluding it from species-specific or clade-specific subsets. In addition, this suggests the possibility of missing GO terms from our subsets that describe such interactions due to the absence of assignments with experimental evidence codes, which again calls for the significance of curatorial expertise accompanying this method.
6. A way that we used to expand our GO Big plant subset was by considering taxon constraints, a correction system that includes the two relationships “only in taxon” and “never in taxon” to help curators flag inconsistencies in annotations [25] and is implemented in tools like the Phylogenetic Annotation Inference Tool (PAINT) and Argot2.5 web server [19, 25]. Indeed, this method was helpful for capturing GO terms that describe functions that are “only in” Viridiplantae, especially those that were not manually assigned to plant genes in the GOA database. On the other hand, not only were there some errant GO terms that were inherited in our GO Big plant subset that are ancestor terms of those describing plant functions as previously described in our results, but some of these ancestor terms were labelled with the “never in" Viridiplantae taxon constraint. This presented a problem as the removal of such terms would lead to the exclusion of entire areas in the DAG. For example, the exclusion of “epidermis development" (GO:0008544), which is labelled with the “never in" Viridiplantae taxon constraint, from our subset would lead to the subsequent exclusion of the child term “epidermal cell fate specification" (GO:0009957). This child GO term has been manually assigned to several genes (*GL2*, *BHLH2*, *GL3*, *TOP6A*, *TTG1*, and *WRKY44*) found in *Arabidopsis thaliana* that play a role in determining the cell fate of root epidermal cells [50–54]. Using taxon constraints alone is not sufficient, and we suggest that these ancestor terms be revised to either become taxon-neutral or provide plant function-equivalent terms.

Future work entails updating the GO Big plant subset with newer file versions possibly by (1) collecting GO terms through curatorial knowledge regardless of evidence code, (2) including all taxon-neutral GO terms, and (3) creating subset variations in which GO terms from certain types of relationships are either included or excluded (as an example, choosing not to include GO annotations with “regulates" relations to avoid the integration of functions that describe interactions affecting other organisms).

### How to GO Big

In this paper, we discussed creating GO Big with the ultimate goal to improve predicted gene function assignments in plants. By following this methodology, researchers can create GO Big subsets tailored to their specific taxon of interest. A practical application for these subsets is using them in tools like GOTermMapper [9, 34] to map errant GO terms, helping to reveal potential gene functions and inspire new hypotheses to test. An important feature for GO Big subsets is that, like GO, they are DAGs. This paves the way for a second application for GO Big: researchers and curators can identify areas of improvement within the graphs for missing or more inclusive function descriptions. These findings can be reported directly to the GO Consortium (https://geneontology.org/docs/contributing-to-go-terms/), as we have done for the GO terms with the non-plant functions that were discussed previously in the results section. Finally, a potential application of GO Big subsets is their integration into gene function prediction tools and GO enrichment analysis tools as an alternative to the full GO DAG, allowing researchers to obtain precise annotations relevant to the species’ biology without concerns about errant GO terms.

### Why should you GO Big?

While the direct purpose of GO Big subsets is to obtain biologically meaningful annotations for the taxon of interest during gene function prediction, there is a larger implication. By restricting annotation assignments to functions valid to the biology of the organism, bias against the assignment of errant functions can be eliminated, allowing for a possible shift in research focus from highly-studied genes to poorly-characterized or unknown genes that have been electronically assigned with functions of interest. In other words, GO Big subsets may drive the advancement of science by indirectly addressing the problem of the significant number of unknown genes in different organisms. It is worth mentioning that considering gene function annotations derived from GO Big subsets along with the unconventional function predictions assigned to the same gene by the full GO DAG may spark new ideas about the possible function of the gene and inspire researchers to generate novel hypotheses to test, so even these annotations can be useful.

## Conclusion

The abundance of sequenced plant genomes, the availability of the largest knowledgebase for gene functions, and the advancement in gene function prediction tools set up the right time and circumstances for the formation of the GO Big subset. In addition, the specific need for the GO Big plant subset arose from the comparatively lesser detail in knowledge of plant functions. By creating a new type of GO subset specifically curated for plants, we hope that this subset would be used by both function prediction tools and GO mapping tools to increase taxonomic unit specificity while restricting to biologically meaningful functions in plants. We also hope that the approach would be adopted and applied to other species resulting in more GO Big subsets. We expect the involvement of more biologists in generating such subsets would be critical in contributing to the GO Consortium’s mission in developing a comprehensive and inclusive model of biological systems.

## Additional file

Additional file 1: Mappings of the 28 GO term annotations for the *Zea mays* B73v5 *sdg130* gene assigned by GOMAP using GOTermMapper. goslim_plant: Excel sheet showing the resulting GO terms when the 28 GO annotations were mapped to the GO Slim plant subset. gobig_maize: Excel sheet showing the resulting GO terms when the 28 GO annotations were mapped to the GO Big maize subset. gobig_plant: Excel sheet showing the resulting GO terms when the 28 GO annotations were mapped to the GO Big plant subset. Rows in orange in the gobig_maize and gobig_plants sheets indicate that the terms are also present in the goslim_plant sheet. Rows in blue in the gobig_plant sheet indicate that the terms are also present in the gobig_maize sheet, but not in the goslim_plant sheet.

Additional file 2: BiNGO output for GO overrepresentation assessment of the 27 *Zea mays* B73v5 *sdg* gene models that contains information about the nodes, edges, and p-values of the GO terms in the generated networks. Each sheet within the file is named after the ontology that was used.

Additional file 3: Figure showing the network of overrepresented GO terms for the 27 *Zea mays* B73v5 *sdg* gene models based on the GO Slim plant subset that was generated by BiNGO.

Additional file 4: Figure showing the network of overrepresented GO terms for the 27 *Zea mays* B73v5 *sdg* gene models based on the GO Big maize subset that was generated by BiNGO.

Additional file 5: Figure showing the network of overrepresented GO terms for the 27 *Zea mays* B73v5 *sdg* gene models based on the GO Big plant subset that was generated by BiNGO.

Additional file 6: Figure showing the network of overrepresented GO terms for the 27 *Zea mays* B73v5 *sdg* gene models based on the full GO DAG that was generated by BiNGO. Legend for Additional files 3-6.pdf: White nodes indicate parent GO terms for overrepresented terms, but are not themselves significant. Yellow nodes indicate statistically significant GO terms (p<0.05), with nodes becoming more orange as significance increases. Node size is determined by the number of genes associated with the GO term.

## Data Availability

All data and source code generated are freely available at https://github.com/Dill-PICL/gobig.git [26] under the terms of the MIT license.

## Code availability

All data and source code generated are freely available at https://github.com/Dill-PICL/gobig.git [26] under the terms of the MIT license.
