# Supplementary figures and images for: Go Big or go home: a new gene ontology subset that improves plant gene function prediction
Source: Plant Methods. 2026 Mar 29;22:46. doi: 10.1186/s13007-026-01523-8 (PMC13154503; doi:10.1186/s13007-026-01523-8)

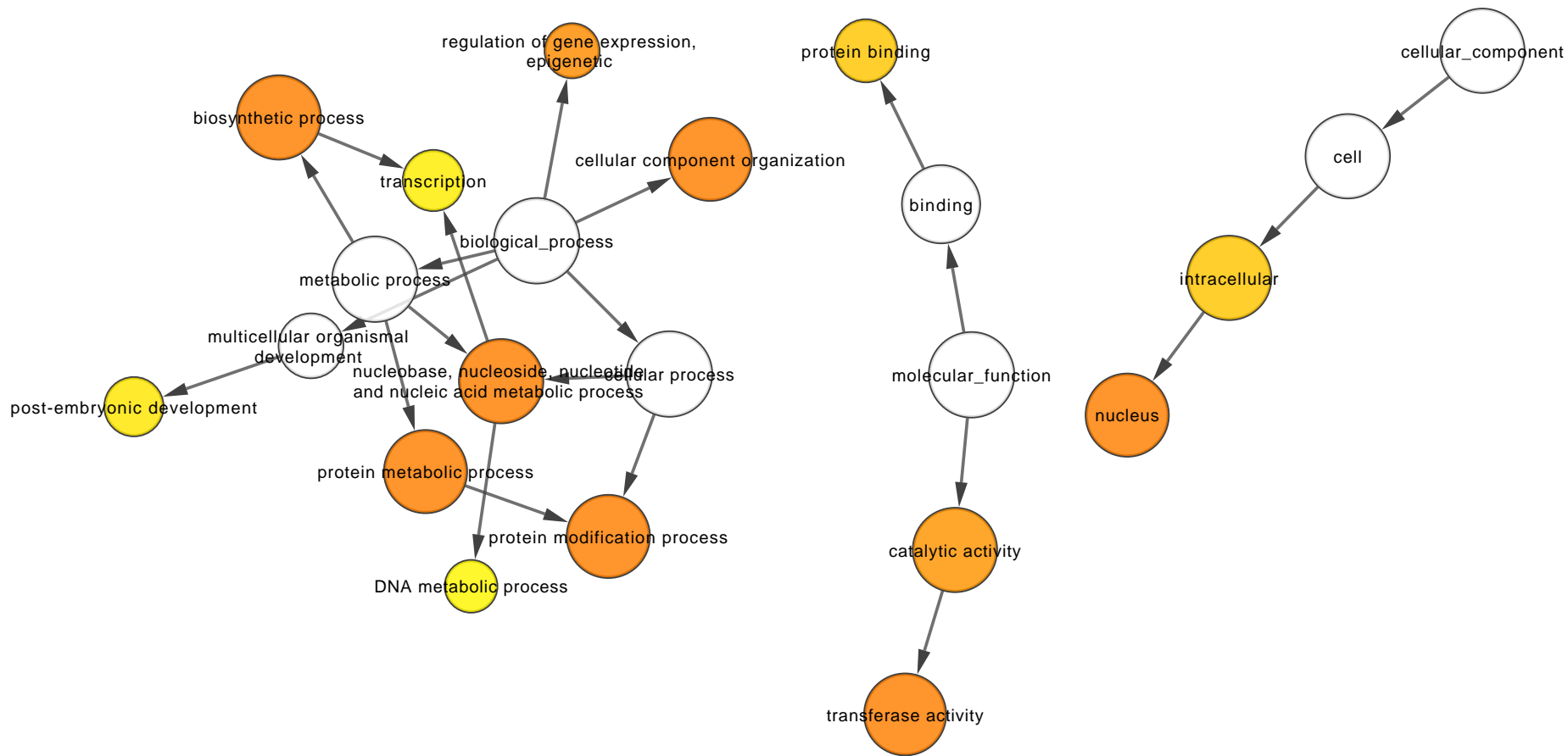

Supplement: Supplementary file 3 — Additional file 3: Figure showing the network of overrepresented GO terms for the 27 Zea mays B73v5 sdg gene models based on the GO Slim plant subset that was generated by BiNGO. [file 13007_2026_1523_MOESM3_ESM.pdf]

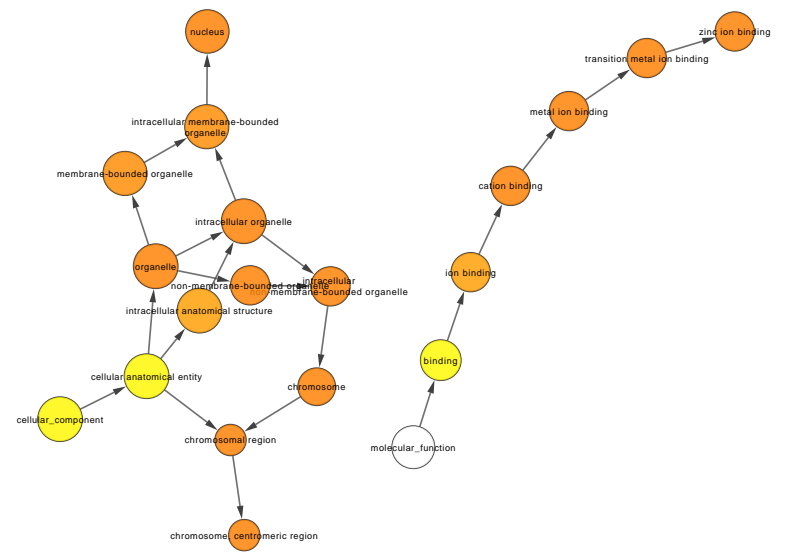

Supplement: Supplementary file 4 — Additional file 4: Figure showing the network of overrepresented GO terms for the 27 Zea mays B73v5 sdg gene models based on the GO Big maize subset that was generated by BiNGO. [file 13007_2026_1523_MOESM4_ESM.pdf]

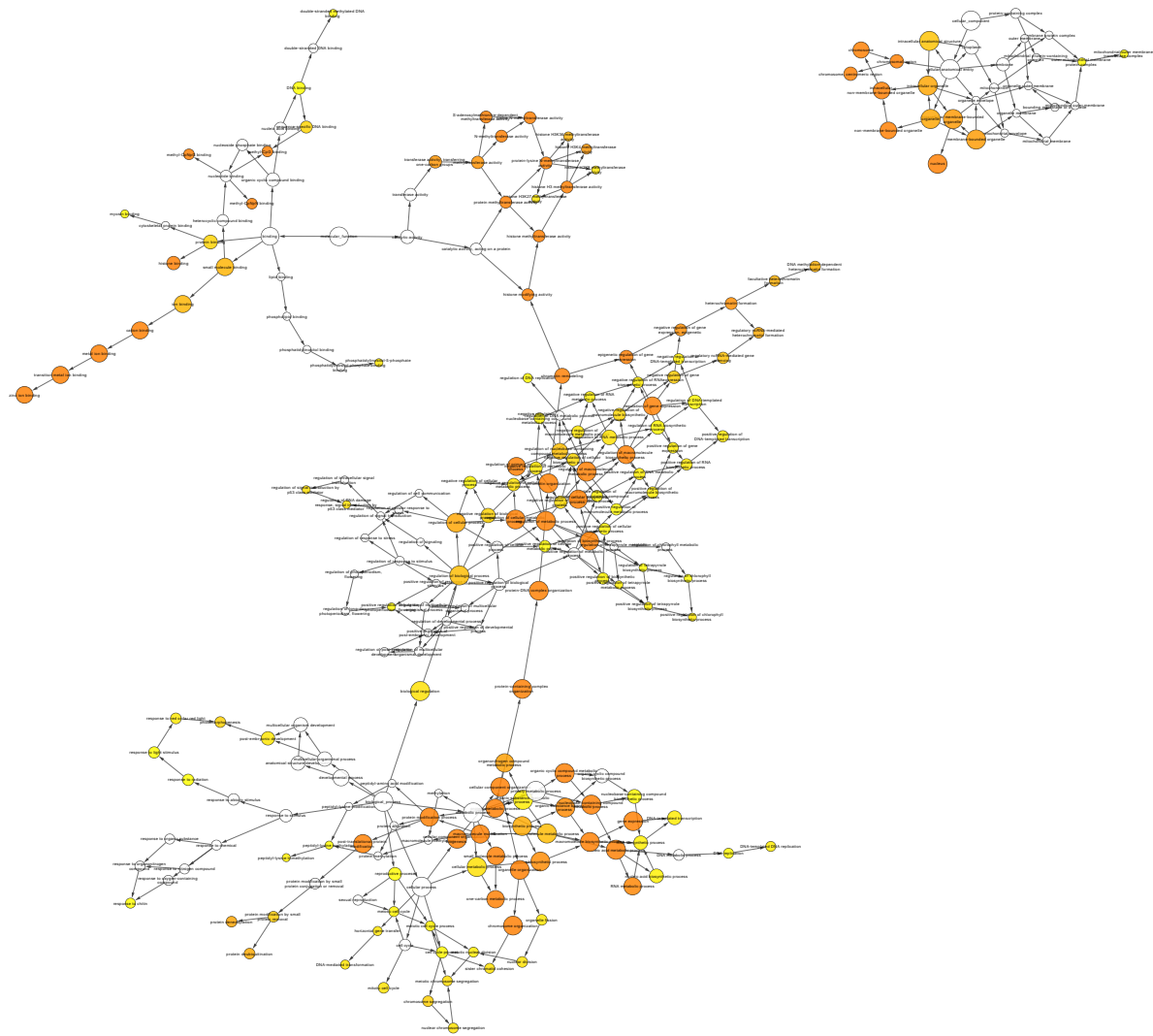

Supplement: Supplementary file 5 — Additional file 5: Figure showing the network of overrepresented GO terms for the 27 Zea mays B73v5 sdg gene models based on the GO Big plant subset that was generated by BiNGO. [file 13007_2026_1523_MOESM5_ESM.pdf]

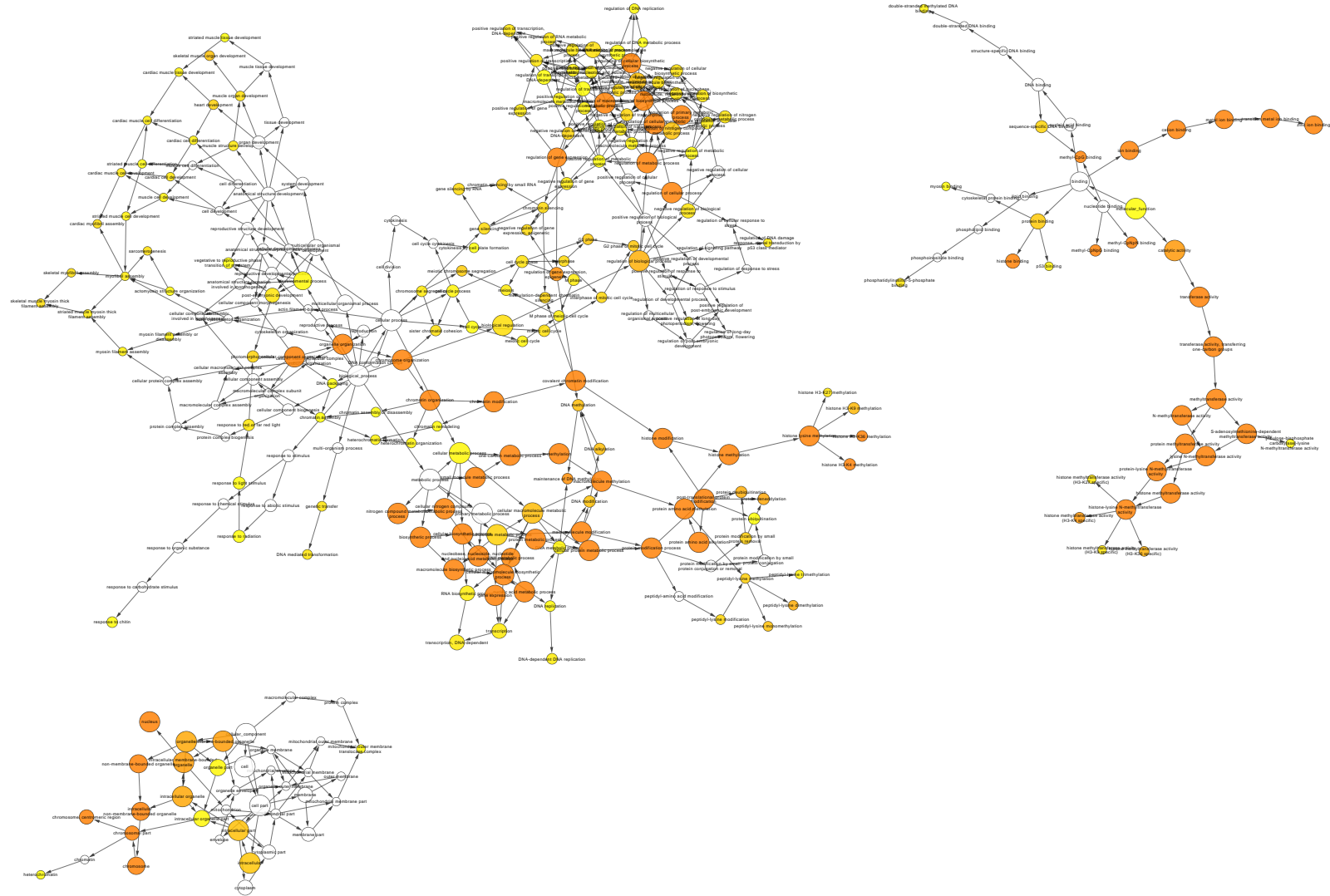

Supplement: Supplementary file 6 — Additional file 6: Figure showing the network of overrepresented GO terms for the 27 Zea mays B73v5 sdg gene models based on the full GO DAG that was generated by BiNGO. Legend for Additional files 3-6.pdf: White nodes indicate parent GO terms for overrepresented terms, but are not themselves significant. Yellow nodes indicate statistically significant GO terms (p<0.05), with nodes becoming more orange as significance increases. Node size is determined by the number of genes associated with the GO term. [file 13007_2026_1523_MOESM6_ESM.pdf]
